# Prediction and Understanding of Resilience in Albertan Families: Longitudinal Study of Disaster Responses (PURLS) – Protocol

**DOI:** 10.3389/fpsyt.2019.00729

**Published:** 2019-10-31

**Authors:** Dawn Kingston, Muhammad K. Mughal, Muhammad Arshad, Igor Kovalchuk, Gerlinde A.S. Metz, Katherine Wynne-Edwards, Suzanne King, Shui Jiang, Lynne Postovit, Abdul Wajid, Sheila McDonald, Donna M. Slater, Suzanne C. Tough, Katherine Aitchison, Paul Arnold

**Affiliations:** ^1^Faculty of Nursing, University of Calgary, Calgary, AB, Canada; ^2^Mathison Centre for Mental Health Research and Education, Hotchkiss Brain Institute, Cumming School of Medicine, University of Calgary, Calgary, AB, Canada; ^3^Biological Sciences Department, University of Lethbridge, Lethbridge, AB, Canada; ^4^Department of Neuroscience, University of Lethbridge, Lethbridge, AB, Canada; ^5^Faculty of Veterinary Medicine & Hotchkiss Brain Institute, University of Calgary, Calgary, AB, Canada; ^6^Department of Psychiatry, McGill University, Montreal, QC, Canada; ^7^Departments of Psychiatry and Medical Genetics, University of Alberta, Edmonton, AB, Canada; ^8^Department of Oncology, University of Alberta, Edmonton, AB, Canada; ^9^Child Development Centre, University of Calgary, Calgary, AB, Canada; ^10^Department of Physiology and Pharmacology, Cumming School of Medicine, University of Calgary, Calgary, AB, Canada; ^11^Department of Obstetrics and Gynaecology, Cumming School of Medicine, University of Calgary, Calgary, AB, Canada

**Keywords:** stress, resiliency, genetics, protocol, child development

## Abstract

Exposure to a natural disaster in childhood can have serious, long-lasting consequences, impacting physical and mental health, development, and learning. Although many children experience negative effects after a disaster, the majority do not, and what differentiates these groups is not well understood. Some of the factors that influence disaster-related outcomes in the midst of adversity include parents’ mental health, the home environment, and socioeconomic status. Furthermore, genetics has also a role to play in how children respond to stressors. We had the opportunity to conduct a natural experiment of disaster recovery following the Alberta 2013 Flood. This paper presents the detailed protocol on prediction of resilience in Albertan families, and validation with cortisol data. In addition, data collection procedures, developing resiliency screening tools, candidate gene identification, genotyping, DNA methylation, and genomic analyses are described to achieve the research objectives. This study produced new knowledge by using pre- and post-disaster information on children’s health and development, including children’s genetics and responses to stress. This information has been identified as important to governments and other organizations invested in early child development. Our comprehensive research plan generates evidence that can be mobilized population-based approaches to improve child and family resiliency.

## Introduction

Population-based cohort studies provide vital information to examine and identify etiological factors in the development of diseases important for public health (1). Cohort studies are not conducted specifically on a diseased population, and hence they provide a comprehensive understanding of environmental factors and their impact before the development of diseases. Undertaking prenatal and postnatal cohort studies is particularly important for investigating the origin of diseases believed to have their origins in the *in utero* environment (2–4). Negative life events can have an impact at a number of points in child development, including the impact of maternal stress *in utero* (5), and children experiencing stress due to natural disaster are more vulnerable to mental health conditions such as depression, anxiety, behavioral, and learning problems (6–8). These stressors may induce epigenetic changes, and also increase risk for later development of adverse mental health outcomes (9). Recently, a study has shown that high stress levels in mothers’ increase the likelihood of epigenetic changes in the child’s genome (10). The importance of cohort studies in linking early events to later life outcomes has been well established, and this may help in identifying potential risks at early stage, as well as in developing preventive measures for the well-being of individuals and communities (11, 12).

## The Issue: Prenatal Stress and Early Adverse Childhood Experiences May Have Serious and Enduring Consequences

The long-term impact of adverse childhood experiences (ACEs) on physical and mental health into late adulthood is well-established (13). Children exposed to natural disasters have higher rates of depression (18–25%), anxiety (12–41%), post-traumatic stress disorder (PTSD) (5–57%) (14–16) behavioral disorders (29%), and developmental/learning problems (29%) (8) than those not exposed. The chronicity of symptoms in this group is particularly concerning, with one-third of exposed children continuing to report symptoms (8, 15) or meet Diagnostic and Statistical Manual of Mental Disorders (DSM) criteria for a mental illness 1 to 3 years post-disaster (17). Recent Canadian studies have shed critical light on the extent of the influence of prenatal exposure to natural stressors including asthma, obesity, and epigenetic changes from early childhood to 13 years (10, 18–25). The consequences of exposure of children to natural disasters are of great public health concern, exerting significant burden on health and education sectors.

## Which Children Are At Risk?

The most studied child outcome, related to disaster exposure, is psychopathology. Few studies have explored risk factors associated with post-disaster outcomes, and they are limited by an inability to control for pre-disaster influences. However, disaster research consistently demonstrates the increased risk of post-disaster psychopathology in children of mothers with mental health problems (25–28). Indeed, studies have found that maternal mental health is a better predictor of post-disaster child mental health and behavior than disaster exposure (14, 29). These findings suggest that maternal mental health is a central factor in whether a child develops mental health problems post-disaster, and importantly, one of the potentially modifiable risk factor identified to date (29, 30). Results from Project Ice Storm indicate that prenatal stress from a natural disaster is linked to epigenetic effects in adolescents demonstrating an enduring impact on the child’s epigenome (10). Only a few studies have explored the role of low family resilience (Family Resilience Measure) (31) and family dysfunction (14, 32) as risk factors for children developing mental health and behavior problems post-disaster, but this evidence is equivocal. However, it is evident that family functioning worsens in disaster-affected families, and that these changes are clearly linked to poor parental mental health (14).

## Genetics Play a Role in Child Resilience to Stress

Childhood trauma is a type of stressor. In general, stress has acute, delayed, and long-term effects on the body (33), and can be classified into “good stress,” “bearable stress,” and “toxic stress” (34). “Good stress” can be coped with by physiological mechanisms, encouraging healthy growth; “bearable stress” states may eventually be turned into homeostasis through successful interventions; whereas, “toxic stress,” which is characterized by the prolonged or frequent activation and dysregulation of stress response pathways, induces long-term changes and damage not only in the brain but also in the whole body (35, 36). For example, it was reported that psychological stress is associated with significantly higher oxidative stress and at least one decade of additional aging in women (37).

In response to stress, stimulation of the amygdala leads to activation of the hypothalamic–pituitary–adrenal (HPA) and sympathetic-adrenal medullary (SAM) axes (36, 38–40). This involves numerous mediators of stress, including: the neurotransmitters glutamate, noradrenaline, serotonin (41), and γ-aminobutyric acid (GABA); neuropeptides such as corticotropin-releasing factor (CRF) and the CRF family of peptides such as urocortins, orexin (42), neuropeptide Y (43), dynorphin (44), and oxytocin; and various stress-related hormones such as cortisol. These mediators bind to neuronal and non-neuronal receptors throughout the body with resultant downstream effects.

Dopamine has been associated with the stress response, and dopaminergic signaling plays important roles in fear extinction (especially in hippocampal area CA3 and the medial prefrontal cortex) (45) and is also relevant to reinforcement learning, motor control, motivation, and mood (46). Dopamine receptors belong to the G-protein coupled receptor (GPCR) family. Dopaminergic neurons can be stimulated or spontaneously activated in various patterns or modes of firing (such as bursts), with different modes being associated with different behaviors (47, 48). Indeed, alterations in the dopaminergic system have been linked to psychosis (49), anxiety disorder (50), depression (49), addiction (51), and autism (52).

The *DRD2* gene on chromosome 11q23.2 encodes the dopamine D_2_ receptor (D2R), which is able to interact with other GPCRs (53). There is a single nucleotide polymorphism (SNP), rs1800497, a C > T substitution is located 10 kb downstream of *DRD2* and leads to an amino acid substitution (p.Glu713Lys) in a gene (*ANKK1*) on the complementary DNA strand (54). The T/T (A1/A1) genotype has been associated with reduced *D2R* availability in the striatum (55), and with addictions (56), mood disorder (57), antisocial behavior (58), and gambling (59). Conversely, the C/C (A2/A2) genotype has been linked to greater vulnerability to depression after stressful life events (60).

The dopamine transporter, encoded by *SLC6A3* (also known as *DAT1*) on chromosome 5p15.33, mediates the reuptake of dopamine from the synaptic cleft (61). Variants in *DAT1* have been associated with attention deficit hyperactivity disorder (ADHD) (62), Tourette’s Syndrome (63), childhood Parkinsonism–dystonia (64), substance abuse (65), and eating disorders (66).

There is a variable number tandem repeat (VNTR) in the 3′ untranslated region of *DAT1*, varying from 3–11 repeats of a 40-basepair element, with the 9 and 10 repeats being the most common (67, 68). Ten-repeat carriers express higher levels of *DAT1* than 9-repeat carriers (68). Under conditions of adversity such as prenatal smoking exposure (69) and psychosocial trauma (70), the *DAT1* 10-repeat has been associated with ADHD symptoms (62).

The catechol-*O*-methyltransferase enzyme encoded by the *COMT* gene on chromosome 22q11.2 (71). Low, intermediate, and high levels of COMT enzyme activity were first identified in red blood cells (72, 73). A SNP in *COMT*, rs4680 (G > A), results in a valine (Val) to methionine (Met) substitution in the enzyme (74). The Val variant is associated with 3–4 fold higher enzyme activity than the Met, and thus a lower dopamine level in the prefrontal cortex (74, 75). Colloquially, this has been termed the warrior (G: lower dopamine level, less vulnerable to stress) versus worrier (A: higher dopamine level, more vulnerable to stress) variant (76).

The serotonin transporter is encoded by *SLC6A4* (also known as the *5-HTT* gene), on chromosome 17q12. At 1 kb upstream is the *5-HTT* linked polymorphic region (*5-HTTLPR*), consisting of a 20 to 23 base pair (bp) GC-rich region which is repeated 14 times in the short allele (S) and 16 times in the long allele (L). The short variant of the polymorphism is associated with reduced *5-HTT* expression (77), with S/S genotype being associated with increased risk of depression and suicide attempts after stressful events (78). An A > G SNP (rs25531) in the *5-HTTLPR* appears to be associated with altered expression levels, such that on the L allele (L_G_), the L then behaves like an S (79).

*In utero* exposure to natural adversities can lead to epigenome changes that can affect developmental and adaptation aspects in children. DNA methylation is a well-studied mechanism of epigenetic, which impacts *utero* stress (80). Furthermore, studies have shown that environmental factors can alter patterns of DNA methylation resulting into long term changes in gene function (81). Similarly, stress reactivity is typically measured by activation of the hypothalamic pituitary adrenal (HPA) system through salivary cortisol (82). Cortisol stress response can be triggered from the nature of stimulus and individual’s perception (83). Anger predicted rise in salivary cortisol and its levels correlated with anger intensity in children (84). Studies have found some similarities and differences in physiological stress responses in boys and girls (85). These differences are believed to result from differential biological mechanisms and physical and psychosocial expression of stress.

Above observations suggest that genetic analyses involving these genes, and identification of new genes associated with stress response, may help in improving our understanding of child and family resilience to natural disaster.

## Resilience: Why Do Some Children Experience Serious Consequences Post-Disaster, and Others Do Not?

Resilience is the ability to adapt or “rebound from adversity when one’s ability to function has been impaired” (86). The most pressing unanswered question in child outcomes research has been why some children do and other do not experience adverse outcomes. The majority of child outcomes research examines risk without resiliency, thus identifying a broad group of children who may develop morbidities is essential. To unravel resilience and attached risks in a longitudinal cohort study, the child’s status before and after the adversity need to be identified in order to understand the child’s response to adversity. To date, contemporary worldwide disaster research exploring children’s outcomes has been unable to tackle this question due to unavailability of pre-disaster data.

## Challenges of Service Provision Post-Disaster

Recent disasters show that child mental health services are unable to address the breadth of services required from prevention to specialized services post-disaster (16). In addition, the increased burden on the healthcare system is prolonged, lasting up to one-year post-disaster (87). While stepped care service provision models have been found to be effective post-disaster, they require identification of children “at risk,” the level of severity of symptoms, and decision pathways that create appropriate linkages between need and service. Mcdermott et al. (16) noted that in response to lack of service capacity, services require new processes to facilitate assessment of children and adolescents (16).

One in seven children requiring mental health services receives them post-disaster, and a recent chart review of children receiving mental healthcare services 3 years post-Katrina reported multiple missed opportunities for early identification and intervention (8). Furthermore, evidence following Hurricane Katrina demonstrated that mental health functioning deteriorated markedly at follow-up assessments for children who did not access services (88). Efficient use of post-disaster resources relies upon accurate identification of children with sub-optimal resilience and efficiently linking those children with effective and acceptable services appropriate to their needs (89).

There is a paucity of knowledge about the relationship between parental resilience and disaster-related outcomes in children (31, 32). Inherent in the identification of at-risk children and consistent with a population-based approach to resiliency is the need to identify the impact of families with low resilience post-adversity. Given that the cohort study has emerged as a popular study design for investigating the gene-environment interactions for public health (1), this design was used in our Prediction and Understanding of Resilience in Albertan Families: Longitudinal Study of Disaster Responses PURLS to describe the effects of natural disaster (Calgary flood 2013) stress on the All Our Families (AOF) in Calgary, Alberta, Canada. The aims of this study include: 1) address the gaps that exist in post-disaster outcomes due to unavailability of pre-disaster data 2) widely disseminate the child resilience screening and triage tools for future use and evaluation 3) validate the resilience screening tools using biological data and 4) promote and guide further research in early prevention/intervention aimed at improving child and family resilience and strengthening protective factors.

## Methods

### The All Our Families (AOF) Cohort Population

The cohort is representative of the pregnant and parenting population in a Canadian urban center (90). The AOF cohort provides longitudinal information on preconception and prenatal experiences, birth outcomes, maternal mental health during the perinatal and early childhood periods, and child health and development outcomes up to 5 years of age.

#### Pre-Disaster Data

Approximately 3,200 women are recruited from health care offices, communities, and through a city-wide, single provider laboratory service in Calgary, Alberta (**Table 1**). In brief, 78% of participants are Caucasian, 22%, non-Caucasian, mean age at delivery is 31 years, and 50% are pregnant with their first child. At the time of the flood (June 2013), ∼450 children in the AOF cohort are between the 12 and 24-month assessments, ∼950 are between their 24 and 36-month assessments, and ∼1,400 are between their 36-month and 5-year assessments. Women are recruited at <25 weeks’ gestation between August 2008 to December 2010. Women complete self-report questionnaire twice during pregnancy and at 4, 12, 24, and 36 months postpartum. The questionnaires include standardized tools and content specific items as described in Mcdonald et al. (90).

**Table 1:** Demographics of pre-flood AOF participant recruitment.

	Demographic characteristics	n/%
1	Total recruited women	3,200
2	Period of gestation	<25 weeks
3	Caucasian	78%
4	Non-Caucasian	22%
5	Women’s average age	31 years
6	Women having first child	50%

#### Post-Disaster Data

The PURLS resilience study is conducted on the participants that are recruited out of the AOF cohort. People who complete the 2013 flood experiences survey in the AOF cohort are invited to join PURLS study. The flood impact survey (**Supplementary Materials**), is administered 6 months post-flood. The flood impact questionnaire for children (**Supplementary Tables 1, 2**), was administered in 2015–2016. The average age of children is 3 years, and range varied form 1–4 years at the time of flood. Consent forms (**Supplementary Table 3**) are sent to AOF in an email through a web link, and the families who consent to participate in the survey are subsequently sent an electronic survey to determine child resilience.

**Figure 1:**
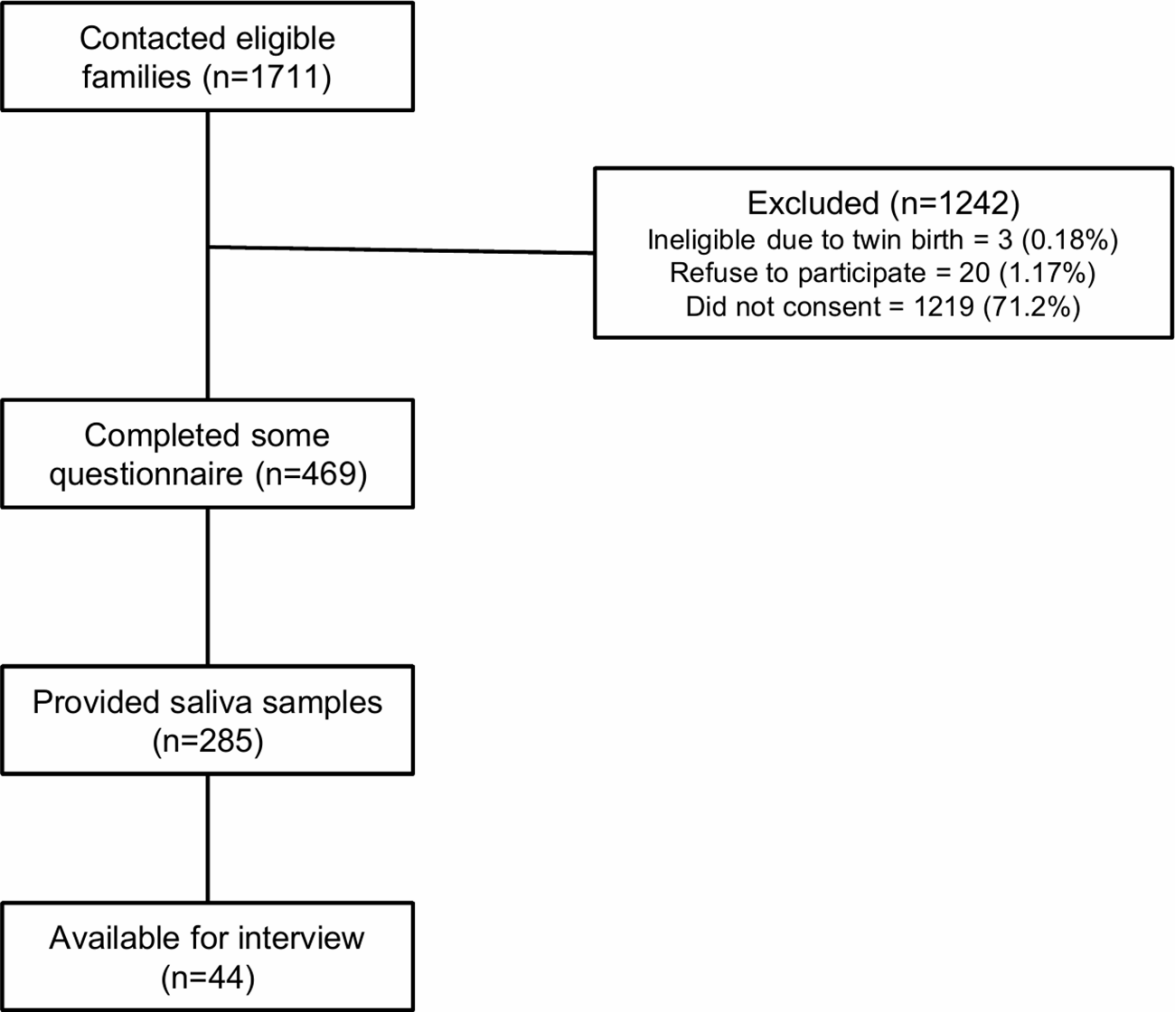
Post-flood AOF participant recruitment.

Of the total 1,711 participants that complete the 2,013 flood survey and are contacted for participation, 23 are excluded due to ineligibility or disinterest in participation (**Figure 1**). Out of the remaining 1,688 participants, 469 (28%) people participate by completing all or some of the electronic resilience questionnaire. A total 285 participants also consent to complete the saliva collection part of the study, and 44 participate in a telephone interview about their family experiences of the flood and their child’s resilience (**Figure 1**).

### Development of Resiliency Screening Tools

**Figure 2:**
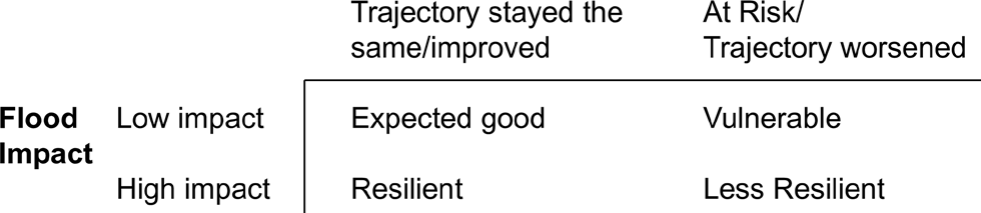
Pattern of pre- and post-flood functioning.

Resilience status is derived by constructing a variable by cross-classifying flood impact with children’s pre-flood (baseline) and post-flood functioning in order to identify baseline status and changes occurring following the flood. Pre- and post-flood functioning is assessed by widely used tools with demonstrated psychometric properties. Specifically, internalizing and externalizing behaviors are measured using the Brief Infant Toddler Social Emotional Assessment (BITSEA) (age 2), the National Longitudinal Survey of Children and Youth’s Behavior Scales (age 3), and the Behavioral Assessment for Children, Second Edition (BASC-2) (age 5), Devereux Student Strengths Assessment (DESSA) and the Devereux Early Childhood Assessment for Preschoolers Second Edition (DECA-P2). The Ages and Stages Questionnaire, 3rd Edition (ASQ-3) assesses children’s cognitive, emotional, social, and motor functioning at all ages. Using scoring algorithms and established clinical cut-offs for these measures, we identify patterns of pre-post functioning i.e. trajectory of functioning worsened (−1SD), stayed same or improved (+1SD), which is combined with flood impact level to identify children’s resilience status (**Figure 2**). Maternal reported child healthcare utilization is used to identify poor mental functioning (e.g. visits to psychologist).

Potential factors associated with child resilience status include child factors (child gender, gestational age, early developmental functioning, temperament), maternal factors (maternal adverse childhood experiences, maternal mental health, parenting competence, and stress), and family/community factors (e.g. SES, child care, social support). “Low” family resilience is defined as a decline in quality of partner relationship or parenting style (e.g., more angry, hostile) post-disaster compared to pre-disaster.

Descriptive statistics is used to describe the sample using frequencies, proportions, means (SD)/medians, IQR) as appropriate. Bivariate and multivariable analyses are conducted followed by Latent Transition Analysis (LTA) to describe how children’s resilience status changes over time. Classification and Regression Trees (CART) analysis is used to determine children’s resilience status according to different combinations of risk factors that were identified in previous analyses (e.g., LTA). These analyses are performed for each separate construct (e.g., behavior, health service utilization) and for composite low resilience, operationalized as classification as low resilient in any of the separate constructs. Separate analysis is performed for low and high risk exposure (impact) (91).

Using a regression coefficient-based scoring algorithm applied to the final predictive regression model for composite low resilience, a screening tool is developed for low child resilience post-disaster. Receiver Operating Characteristics (92) analysis is applied to determine the sensitivity, specificity, positive, and negative predictive values for different cut‐offs of the screening score, and established cut-offs for mild, moderate, and severe status.

### Biological Sample Collection

All biological samples are obtained from November 2015 to November 2016. Completed kits are picked up from the home by a study employee and returned to the laboratory by mail. The collection include a total of 8 samples *per* child for 285 children: 3 samples *per* day for two days for cortisol analysis (n=6 samples), a DNA sample, and an RNA sample.

Each package contains a kit for DNA and RNA sample collection and sample collection instructions. Back in the laboratory, DNA/RNA samples are separated from saliva samples and immediately stored at −80°C until analyses. Preliminary analyses confirm that the collected DNA is viable basis for a range of genomic and epigenomic applications, ranging from single nucleotide polymorphism genotyping, to microarray analysis, to methylation analysis using a bisulphite protocol.

Using the second package, children are provided six whole saliva samples using age-appropriate Salimetrics collection tubes and child sorbettes (cotton swabs: salimetrics.com). Cortisol samples are collected at home just after awakening, 30 min later, and just before bedtime on two consecutive days. Instructions are to delay toothbrushing until after collection, and to securely hold one end of the swab and place the other end under the child’s tongue as *per* manufacturer’s instructions (Salimetrics LLC, Carlsbad CA).

### Validation of Screening Tool Using Cortisol Data

The field of resilience suffers from limited translation of biological markers of adversity into clinical assessment or intervention. A vital component of this project is the use of such biological markers to validate the resilience screening tool. Distinguishing the biological signatures of children with low and high resilience is thus a complementary approach to validating the definition of the resilience phenotype identified through psychosocial and clinical data. The adrenal steroid, cortisol hormone plays a crucial role in the response to adverse life events, a biomarker of current stress (93–95). Based on Cao et al. (23), 285 children are an adequate sample size for the proposed biological analyses. Resiliency screening tools are used to identify resilient and non-resilient children. Biological samples collected from the AOF children are analyzed for cortisol biomarkers, which is tested for association with resiliency status to biologically validate the resiliency tool. Moreover, genetic and genomic analyses is conducted to identify genes and/or single nucleotide polymorphism (SNPs) in resilience versus non-resilient children.

### Circadian Cortisol Pattern and Repeatability Across Days

Established methods in liquid chromatography coupled to tandem mass spectrometry (LC-MS/MS) (96) are used to quantify the salivary adrenal stress hormone cortisol. Cortisol is purchased from Steroids Inc (Newport, RI). Deuterium labeled internal standard: cortisol-d4, is obtained from CDN Isotopes Inc (Pointe-Claire, Quebec, Canada). HPLC grade methanol, Optima grade acetonitrile and Optima grade water are purchased from Fisher Scientific (Edmonton, AB, Canada). Ammonium fluoride (NH_4_F) is purchased from Sigma-Aldrich (Oakville, ON, Canada).

#### Sample Preparation Procedure

Saliva sample tubes are centrifuged for 20 min at 2,000 ×g, and then 1 ml of each sample is transferred into a 1.5 ml Eppendorf tube. Sample preparation is performed on a Hamilton MICROLAB STAR^LET^ workstation by diluting 75 µl of each sample or calibrator or quality control (QC) with 75 µl of protein precipitation (PPT) solution including the deuterated cortisol internal standard [9 mg/ml ZnSO_4_.7H_2_O Methanol/H_2_O solution (90/10 v/v)]. The mixture is vortexed for 30 s before being incubated for 20 min at 4°C. Samples are then centrifuged at 18,000 ×g for 15 min and 90 µl of supernatant is submitted for quantitation.

##### Lc-Esi/Mrm Analysis

An Agilent 1,200 binary liquid chromatography (LC) coupled to an AB SCIEX QTRAP^®^ 5,500 mass spectrometer equipped with electrospray ionization (ESI) source is used. LC separation uses an Agilent ZORBAX Eclipse plus C18 column (100 × 2.1 mm, 1.8 µm particle size) at 40°C. Mobile phase A is H_2_O/acetonitrile (95/5, v/v, 2mM NH_4_F) and mobile phase B is 100% acetonitrile (2mM NH_4_F). The 12 min gradient is 15–70% B (0–6 min), 70–100% B (6–7 min), 100% B (7–8.5 min), 100–15% B (8.5–9 min) and is then held at 15% B for 3 min. Cortisol and cortisol-d4 are detected by positive LC-ESI/MRM, with qualifier and quantifier transitions and ionization conditions as listed in **Supplementary Tables 4–8**.

##### Quantitation Method

All steroids are quantified as the area ratio relative to the biologically identical internal standard, using a linear calibration curve with 1/× weighting (range 0.02 to 100 ng/ml). All calibration curves (11 runs) are expected to have R^2^ > 0.998. The lower limit of quantitation, defined as the lowest concentration that gives <20% CV and < ± 30% error will be 0.20 ng/ml. Quality control solutions at 5 and at 0.5 ng/ml are included in all runs. QC replicates are expected to have a CV 6–8%, with precision >90%.

##### Saliva Parameters

Any samples (expected to be <5% of approximately 1,800 samples) that fall below the LLOQ have their concentration imputed evenly within the range of 0.1 to 0.2 for subsequent analyses. Cortisol concentrations are used to calculate 12 parameters for each child: global minimum and maximum concentration across six samples, difference between awakening and 30 min after awakening on each day (plus mean, sd, and CV across the two days), and area under the curve for the day (plus mean, sd, and CV across the two days).

Linear regression model is employed for cortisol levels at awakening, morning, night, and awakening response (morning minus awakening) are used as an outcome along with resilience, child sex, flood impact as independent variables. This analysis show if resilience predicts cortisol levels in boys/girls, impacted/not impacted by flood.

### Genetic, Genomic, and Transcriptomic Analyses for Candidate Genes

#### A. Genetic Analysis

##### Candidate Genes Associated With Stress

Candidate genetic markers are chosen by virtue of their involvement in the stress response by the hypothalamus–pituitary axis (HPA) and relevant mental health phenotypes (see *Introduction* section). Genotyping of the few candidate genes associated with stress or resilient is conducted as described below. Genotyping of DRD2 rs1800497 is conducted using a TaqMan^®^ SNP Genotyping Assay on an Applied Biosystems ViiA^™^ 7 Real-Time PCR System (ThermoFisher Scientific, Canada, formerly Applied Biosystems by Life Technologies, Canada). Similarly, genotyping of DAT1 is conducted as previously described, with minor modifications (97), using primer sequences as follows: forward 5′-TGT GGT GTA GGG AAC GGC CTG AG, and reverse: 5′-CTT CCT GGT CAC GGC TCA AGG.

The COMT rs4680 SNP is genotyped using a TaqMan^®^ SNP Genotyping Assay on an Applied Biosystems ViiA^™^ 7 Real-Time PCR System (ThermoFisher Scientific, Canada, formerly Applied Biosystems by Life Technologies, Canada) as previously described (98). Furthermore, *5-HTTLPR* variant is genotyped as previously described, with minor modifications (99), with primer sequences of 5′-ATG CCA GCA CCT AAC CCC TAA TGT (forward) and 5′-GGA CCG CAA GGT GGG CGG GA (reverse).

#### B. Epigenetics

DNA methylation is measured using whole genome bisulfite sequencing, with validation of a subset of identified affected CG sites. DNA undergoes bisulfite conversion with the EpiMark Bisulfite Conversion Kit (NEB, #E3318S) followed by library generation using the NEBNext Ultra II DNA Library Kit and NEB methylated adaptor/primers as *per* manufacture instructions. Each library is sequenced using an Illumina NextSeq sequencer using 75 bp single-end reads. Analysis is conducted at the University of Lethbridge using open-source software packages that enables the quantitative high-resolution analysis of DNA methylation patterns from bisulfite sequencing data.

The analysis is done in two different version; relaxed and stringent. In case of the relaxed version, a CpG region is covered in a minimum 4 samples in each of the experimental groups. In case of the stringent version, the minimum number of samples requirement is increased to 10 in each of the experimental groups. The minimum coverage to estimate percent methylation for CpG position is set at 10 reads. The minimum length of each clustered CpG stretch is set at 1 CpG (i.e. the smallest possible segment of continuous methylation change is a single CpG).

In case of the relaxed analysis, over 20,000 genomic intervals in total could be examined. Intervals with adjusted *p* values <0.05 and methylation difference over 10% is considered significantly changed — differentially methylated regions (DMRs). The results are annotated with closest feature, distance to transcription start site (TSS), feature overlaps, gene symbols, entrez ids, and gene description and overlap with CpG islands and CpG shores. It appears that DMRs tend to be located around TSS, but only a minority of them overlap with promoter areas, and a very few overlaps with CpGs and CpG shores.

Furthermore, we determine if DNA methylation is globally altered in vulnerable versus resilient populations, and also identify loci that is specifically affected. Validation of differential methylation patterns is conducted at University of Alberta using bisulfite sequencing. Briefly, after conducting a bisulfite conversion step, PCR based amplification is performed. PCR product is cloned and sequenced, to reveal differences in methylation at each CG dinucleotide. For these validation studies, 10 regions are analyzed, which are chosen based on the extent of differential methylation and on the probability that the expression of a gene related to stress responses or immunity may be affected by the methylation. We further investigate the degree of correlation between the level of methylation in the child DNA at 5-year follow-up and the cortisol levels in the child.

#### C. Genomic Analysis

The Infinium Global Screening Array (comprising over 640,000 markers) is used with PsychArray add-on (50,000 markers specific for neuropsychiatric disorders) at Deltagenomics (Edmonton AB). Arrays are run in two batches. Validation of array data for two markers (*DRD*2 rs1800497 and *COMT* rs4680) is undertaken by comparison with the above TaqMan genotyping data.

Genome-wide association analysis (GWAS) is undertaken using resilience as the primary phenotype of interest, taking into account variables pertaining to the children (gender, gestational age, early development functioning, and temperament), mothers (adverse childhood experiences, maternal mental health), and families (family resilience, social economic status, child care, and social support).

#### D. Transcriptomic Analysis

Gene expression analysis is conducted using saliva samples and following next generation sequencing (NGS) workflow. RNA is extracted using the Oragene-RNA-RE-100 kit, with PrepIT,LTP (#PT-L2P-5), RE-100-L2N (#RE-L2N-5) according to manufacturer instructions (DNA Genotek, Ontario, Canada) and mRNA libraries are prepared using TruSeq Stranded mRNA Library Prep Kit (Cat. No. RS-122-2101) and following instructions included in the kit. RNA sequencing reactions are performed using flow cell allowing for high output 75 nt single-end reads on Illumina NextSeq500 following manufacturer’s instructions (Illumina, San Diego CA). Initial quality check is performed on the obtained raw sequencing data using FastQC software (v0.11.5) (https://www.bioinformatics.babraham.ac.uk/projects/fastqc/). Adapter is trimmed and low quality reads are removed using Trim Galore with cutadapt (v1.8.dev8) (https://www.bioinformatics.babraham.ac.uk/projects/trim_galore/). Subsequently, sequenced reads are mapped against GRCh37 (Ensemble build) reference genome using bowtie (v.2.1.0.0) genome assembly tool available in Tophat package (v.2.10) (100). Read count is performed with featureCounts (v.1.5.0-p1) (101), and statistical comparisons are conducted using DESeq2 (v1.18.1) in R computing environment (https://www.r-project.org/). Gene expression results that are obtained from NGS data is further validated with quantitative real time PCR (qRT-PCR) (102).

## Results

The PURLS study was funded in 2015, and AOF recruitment and data collection was started in 2016. Physiologically, lack of resilience is most evident in changes to biomarkers related to the stress response, including immune function (cytokines; steroid hormones-cortisol) and metabolomics (trace elements and minerals). Cortisol fluctuations have been associated with exposure to disaster and trauma in children (10, 103). Ensuring the accuracy of the tool is a necessary step prior to implementing it in a disaster situation. A rigorous method of verifying the accuracy of the screening tool is to compare children identified with “low” and “high” resiliency through the screening tool against children identified through the cortisol biomarkers. Results of this study determine 1) the effectiveness of resiliency screening tool for early identification of children mental health, 2) capability of the triage decision algorithm that links the type of need and its severity identified through the resilience tool, 3) association of genetic modification to child resilience, and 4) prospects of employing results from this study in policy making for mental health.

## Discussion

No other contemporary studies worldwide have been able to study child resilience related to disaster rigorously because of the lack of pre-disaster data. Based on existing gaps in the science of resiliency and its integration into practice and policy, the four key areas that are likely to have the greatest impact in terms of generating and translating evidence to practice and policy are: 1) Early identification of at-risk children. Currently, there is no method to identify children’s level of resilience post-disaster in order to triage children appropriately to effective services. For children to access needed services in a timely manner post-disaster, there is a need to develop a screening tool and decision algorithm that would accurately identify children’s risk level post-disaster and efficiently triage them into a stepped-care system. Part of this screen would necessarily entail identifying at-risk families that do not function or parent effectively post-disaster because of the adverse impact this is likely to have on child resiliency, parental capacity to support the child post-disaster, and child outcomes. This study provides needed clinical evidence to implement population-based approaches to improve child resiliency. 2) Understanding the experience of families impacted by disaster. Only one study to date has described parents’ views of and responses to their children’s needs post-disaster (104). There is a need to support the clinical component of early identification and triage of at-risk children with children’s and families’ experiences, responses and needs post-disaster. 3) At present, there are no Canadian guidelines or position statements related to early identification/intervention of resilience in children in order to support population-based prevention activities or post-disaster intervention. Consensus statements are critical for the organization of prevention and intervention strategies, particularly where evidence gaps exist.

## Conclusion

**Figure 3:**
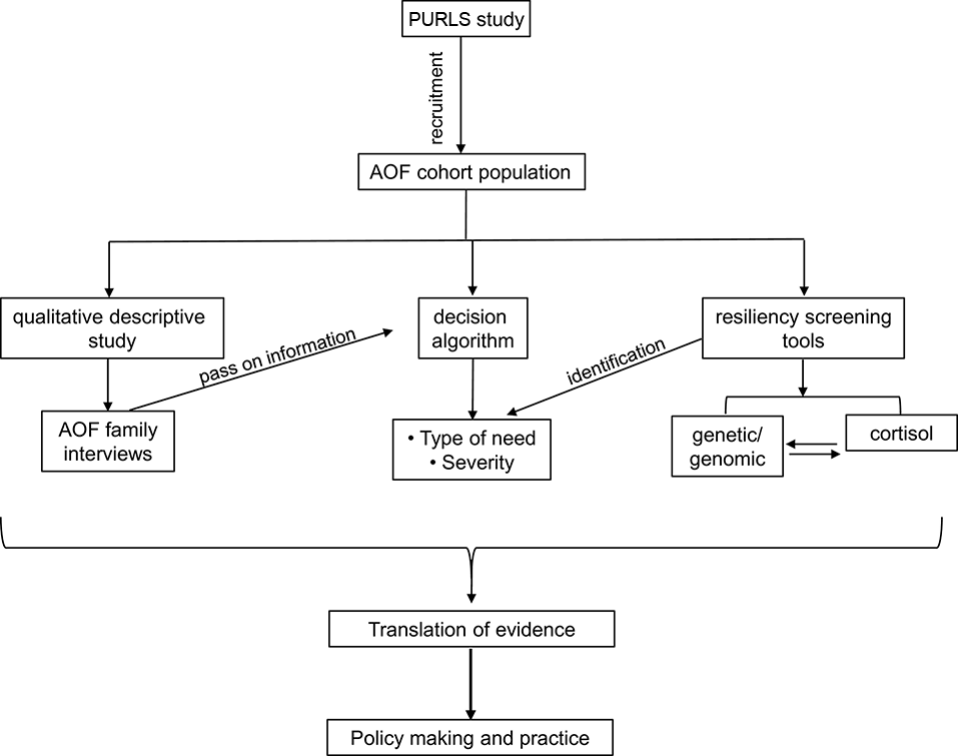
A flowchart showing the PURLS study plan and proposed outcome.

To our knowledge, PURLS is the first study that provides information on pre- and post-disaster data on child resilience. This study contributes to our understanding of methods for identification of resilient and non-resilient children supported by biological information. Identification of genes involved in stress response, associating them to child resiliency and exploring genetic modifications under stress, may enhance our understanding of family and child development. Resiliency screening tool may identify children at risk and their type of need, and this information can be helpful in policy making (**Figure 3**).

## Ethics Statement

This study was carried out in accordance with the recommendations of “Conjoint Health Research Ethics Board, University of Calgary” with signed consent form/consent implied by specific action (Electronic consent) for surveys and interviews from all subjects. The protocol was approved by the University of Calgary Conjoint Health Research Ethics Board (#REB16-0133).

## Author Contributions

DK conceptualized the project. DK, IK, GM, KW-E, SK, KA, LP, SM, PA, ST, and DS designed the project and secured grant funding. MA drafted the manuscript and handled the authors/reviewer’s comments. MM, AW, and SJ provided text for the manuscript. All the authors read and approved the final manuscript.

## Funding

PURLS research study was funded through Alberta Innovates, Grant #201400571. PA receives funding from the Alberta Innovates Translational Health Chair in Child and Adolescent Mental Health.

## Conflict of Interest

The authors declare that the research was conducted in the absence of any commercial or financial relationships that could be construed as a potential conflict of interest.
