# Supplementary material for: Prediction and Understanding of Resilience in Albertan Families: Longitudinal Study of Disaster Responses (PURLS) – Protocol
Source: Front Psychiatry. 2019 Oct 31;10:729. doi: 10.3389/fpsyt.2019.00729 (PMC6834684; doi:10.3389/fpsyt.2019.00729)
Supplement: Supplementary file 1 [file DataSheet_1.pdf]

5807083999

Flood 2013: Impact Survey

Please enter today's date: ___ / ___ / ___
MMM DD YYYY

The 2013 flood in Alberta was a major event for many and we are interested in your family's experience. These findings are important to inform recovery strategies in the future and we greatly appreciate your time in completing this questionnaire.

Thank you again for your ongoing contribution to our research study.

1. What community were you living in at the time of the flood?

Name of community: ______________________

2. What was your postal code at the time of the flood? ___ - ___

3. Have you ever experienced a natural disaster before (e.g., floods, fires, earthquakes, ice storms)?

○ Yes

○ No

The next questions refer to your experiences during the flood. We are interested in understanding how you and your family were affected during the flood.

4. Overall, on a scale of 1-5, how impacted were you by the flood?

○ 1 ○ 2 ○ 3 ○ 4 ○ 5

Not impacted at all — Somewhat impacted — Very impacted

5. Thinking back to June 2013, on a scale of 1-5, how prepared were you for the flood (e.g., groceries in house, financial resources, alternate housing)?

○ 1 ○ 2 ○ 3 ○ 4 ○ 5

Not prepared at all — Somewhat prepared — Very prepared

6. Did you evacuate your household?

○ Yes

○ No (If NO, continue to question 7)

a. If YES, was the evacuation:

○ Mandatory (I was required to leave by official notice - e.g., building manager, police officer, or city blog post)

○ Voluntary (I was given the option to leave or stay by official notice)

○ I decided to leave on my own

b. Did you go to an evacuation shelter?

○ Yes - registered and stayed

○ Yes - registered but did not stay

○ No

Study ID: ___

c. How much time did you have to prepare yourself before you left your household?

- ○ Less than 30 minutes
- ○ 30 - 59 minutes
- ○ 60 - 89 minutes
- ○ 90 - 119 minutes
- ○ 2 hours - 24 hours
- ○ More than 24 hours

d. From when you were evacuated, how long was it until you returned home?

|___|___|___| days

- ○ I have not returned to my original dwelling yet

e. Were you separated from members of your household or them from you when you left?

- ○ Yes
- ○ No

f. Were you separated from pets when you left?

- ○ Yes
- ○ No
- ○ N/A (I do not have pets)

g. Was there a period of time that you were unsure of the condition of your home in terms of loss or damage when you were away from your home?

- ○ Yes
- ○ No

7. The next set of questions asks about damage, loss, injury, and threat as a result of the flood.

a. Was your dwelling damaged? (e.g., house, apartment unit, townhouse)

- ○ Yes
- ○ No

If YES, how would you best describe the damage?

- ○ Could not be repaired (i.e., had to be torn down)
- ○ Damaged - I/we had to move out while repairs were made
- ○ Damaged - I/we stayed in the house while repairs were made

b. Did you experience loss or damage to the following? (check all that apply)

- ○ Neighbourhood
- ○ Community services (e.g., playground, library, school)
- ○ Personal motor vehicle(s)
- ○ Pets
- ○ Personal possessions
- ○ Other: ______________________
- ○ Did not experience loss or damage

c. Did you or a member of your household experience a loss of income?

- ○ Yes
- ○ No

d. Did you suffer a loss of business income?

- ○ Yes
- ○ No
- ○ N/A

e. Did you or a member of your household experience a job disruption as a result of the flood?

- ○ Yes
- ○ No
- ○ N/A

f. Were you injured as a result of the flood?

- ○ Yes
- ○ No

g. Was a close friend or family member injured as a result of the flood?

- ○ Yes
- ○ No

h. Were you ever in danger as a result of the following? (check all that apply)

- ○ Moving waters
- ○ Electrical threats (e.g., exposed wires, electrical power lines)
- ○ Exposure to carbon monoxide
- ○ Lack of drinking water
- ○ Lack of food
- ○ Floating debris
- ○ Was not in danger

8. How many days passed until you felt your life was "back to normal" or in a regular routine?

|__|__|__| days ○ Life is not "back to normal" yet

9. In general, did you feel you were kept up-to-date and informed with events involving the flood?

○ Yes ○ No

Now we would like to ask you about your experiences with flood relief and aid distribution. This will help us to understand how these efforts could be improved for future emergency situations.

10. Did you LOOK FOR help/aid because of the flood?

○ Yes ○ No

11. Did you RECEIVE help/aid because of the flood?

○ Yes

○ No (If NO, continue to question 13)

If YES, what help/aid did you RECEIVE and from WHOM? (check all that apply)

Type of help/aid received

From whom?	Emotional/ psychological	Financial	Medical	Information	Practical support (e.g., housing, childcare, clean-up)	Material goods/donations (e.g., furniture, clothing)
Family (spouse, parent, other relatives)	○	○	○	○	○	○
Friend(s)/neighbour(s)	○	○	○	○	○	○
Community/volunteer organization	○	○	○	○	○	○
Professional (doctor, lawyer, teacher, counsellor, spiritual leader, financial advisor)	○	○	○	○	○	○
Media (TV, internet, social media)	○	○	○	○	○	○
Alberta Health Services (Health Link, information on public health issues, Mental Health Help Line)	○	○	○	○	○	○
Government (debit cards, flood "clean-up" information, resources)	○	○	○	○	○	○
Employer	○	○	○	○	○	○

12. The next few questions ask about your perception of received help/aid.

	Strongly Agree	Agree	Disagree	Strongly Disagree
In general, I believe that I received an adequate amount of help and aid	○	○	○	○
Even today, I still feel slighted because I believe I received less help and aid than I should have received	○	○	○	○
I believe I received too much help	○	○	○	○

13. Did you PROVIDE help/aid to others because of the flood?

- ○ Yes
- ○ No (If NO, continue to question 14)

If YES, what help/aid did you PROVIDE and to WHOM? (check all that apply)

Type of help/aid provided

To whom?	Emotional/ psychological	Financial	Medical	Information	Practical support (e.g., housing, childcare, clean-up)	Material goods/donations (e.g., furniture, clothing)
Family (spouse, parent, other relatives)	○	○	○	○	○	○
Friend(s)/neighbour(s)	○	○	○	○	○	○
Community/volunteer organization	○	○	○	○	○	○
Employer	○	○	○	○	○	○

14. The following questions concern your feelings and opinions about other people, especially about how people were feeling toward each other in the <u>first few days after the flood</u>. For the next set of questions, please indicate to what extent you agree or disagree with each statement.

In the first days after the flood...	Strongly Agree	Agree	Neutral	Disagree	Strongly Disagree
People united and prior disagreements and differences among people disappeared	○	○	○	○	○
I felt rejected and neglected by other people	○	○	○	○	○
People were selfish and cared only for themselves	○	○	○	○	○
I felt as part of a united community of people who experienced a shared misfortune	○	○	○	○	○

15. The next set of questions refers to <u>NOW, several months after the flood</u>. Please answer these as they apply to the people that live in your community or in your neighbourhood.

Now, several months after the flood...	Strongly Agree	Agree	Neutral	Disagree	Strongly Disagree
On a day-to-day basis people are nicer toward each other than they were before the disaster	○	○	○	○	○
People are more sincere, honest, and open toward each other	○	○	○	○	○
People in this community are more integrated and united	○	○	○	○	○
People have a stronger sense that we are all part of a community. In other words, people now have a greater sense of solidarity with others	○	○	○	○	○

16. The next questions refer to the time frame of THE PAST SEVEN DAYS only. Below is a list of difficulties people sometimes have after stressful life events (in this case, the recent flood). Please read each item and indicate how distressing each difficulty has been for you DURING THE PAST SEVEN DAYS.

During the past seven days...	0 Not at all	1 A little bit	2 Moderately	3 Quite a bit	4 Extremely
Any reminder brought back feelings about it	○	○	○	○	○
I had trouble staying asleep	○	○	○	○	○
Other things kept making me think about it	○	○	○	○	○
I felt irritable and angry	○	○	○	○	○
I avoided letting myself get upset when I thought about it or was reminded of it	○	○	○	○	○
I thought about it when I didn't mean to	○	○	○	○	○
I felt as if it hadn't happened or wasn't real	○	○	○	○	○
I stayed away from reminders about it	○	○	○	○	○
Pictures about it popped into my mind	○	○	○	○	○
I was jumpy and easily startled	○	○	○	○	○
I tried not to think about it	○	○	○	○	○
I was aware that I still had a lot of feelings about it, but I didn't deal with them	○	○	○	○	○
My feelings about it were kind of numb	○	○	○	○	○
I found myself acting or feeling like I was back at that time	○	○	○	○	○
I had trouble falling asleep	○	○	○	○	○
I had waves of strong feelings about it	○	○	○	○	○
I tried to remove it from my memory	○	○	○	○	○
I had trouble concentrating	○	○	○	○	○
Reminders of it caused me to have physical reactions, such as sweating, trouble breathing, nausea, or a pounding heart	○	○	○	○	○
I had dreams about it	○	○	○	○	○
I felt watchful and on guard	○	○	○	○	○
I tried not to talk about it	○	○	○	○	○

This assessment is not intended to be a diagnosis. If you are concerned about how you are feeling and coping, please speak with a health professional.

17. Below is a list of the ways you might have felt or behaved. Please indicate how often you have felt this way during THE PAST WEEK.

This past week...	Rarely/None of the time	Some/A little of the time	Occasionally/ Moderate amounts of time	Most/All of the time
I did not feel like eating; my appetite was poor	○	○	○	○
I felt that I could not shake off the blues even with help from my family or friends	○	○	○	○
I had trouble keeping my mind on what I was doing	○	○	○	○
I felt depressed	○	○	○	○
I felt that everything I did was an effort	○	○	○	○
I felt hopeful about the future	○	○	○	○
My sleep was restless	○	○	○	○
I was happy	○	○	○	○
I felt lonely	○	○	○	○
I enjoyed life	○	○	○	○
I had crying spells	○	○	○	○
I felt that people dislike me	○	○	○	○

18. Here are 6 statements that people use to describe how they are feeling. Please select the response that indicates how you feel RIGHT NOW (in this moment).

Right now...	Strongly Agree	Agree	Disagree	Strongly Disagree
I feel calm	○	○	○	○
I am tense	○	○	○	○
I feel upset	○	○	○	○
I am relaxed	○	○	○	○
I feel content	○	○	○	○
I am worried	○	○	○	○

19. Now we would like you to compare your physical and mental/emotional health before and after the flood.

	Excellent	Very good	Good	Fair	Poor
In general, would you say your current physical health is:	○	○	○	○	○
In general, would you say your current mental/emotional health is:	○	○	○	○	○

	Better	About the same	Worse
Your current physical health compared to <u>before</u> the flood is:	○	○	○
Your current mental/emotional health compared to <u>before</u> the flood is:	○	○	○

20. Since the flood(s), have you started taking <u>or</u> increased the use of any prescription medication for your emotional or mental health?

○ Yes

○ No

21. Stressful situations have the potential to affect the relationships around you. We understand that many things may have changed in your life due to the impact of the flood. In the next set of questions, we are interested in how your relationships have changed since the flood.

My relationship with...	has become closer than before the floods	is about the same as before the floods	is more distant than before the floods
Intimate partner<br>○ N/A (I do not have a partner)	○	○	○
Other family members (excluding intimate partner)	○	○	○
Neighbours	○	○	○
People you do not know but are in your community	○	○	○

Has anything else happened to you from the flood that has not been covered in this survey or that you would like to share with us?

Thank you!

Tear-off Sheet
(page will be separated after we receive the questionnaire)

If you would like to be entered into the draw for a $35 gift certificate Hudson's Bay Company gift certificate, please indicate below. Note, there will be one certificate drawn for every 50 responses to this questionnaire. Winners will be notified by mail.

○ No, I do not wish to be entered into the draw for a $35 gift certificate

○ Yes, I would like to be entered into the draw for a $35 gift certificate

Name: ______________________

Mailing address:

Suite number   Street address

City   Province   Postal code

Thank you for taking the time to complete this survey!
