# Supplementary material for: Prediction and Understanding of Resilience in Albertan Families: Longitudinal Study of Disaster Responses (PURLS) – Protocol
Source: Front Psychiatry. 2019 Oct 31;10:729. doi: 10.3389/fpsyt.2019.00729 (PMC6834684; doi:10.3389/fpsyt.2019.00729)
Supplement: Supplementary file 2 [file Presentation_1.pptx]

## Slide 1
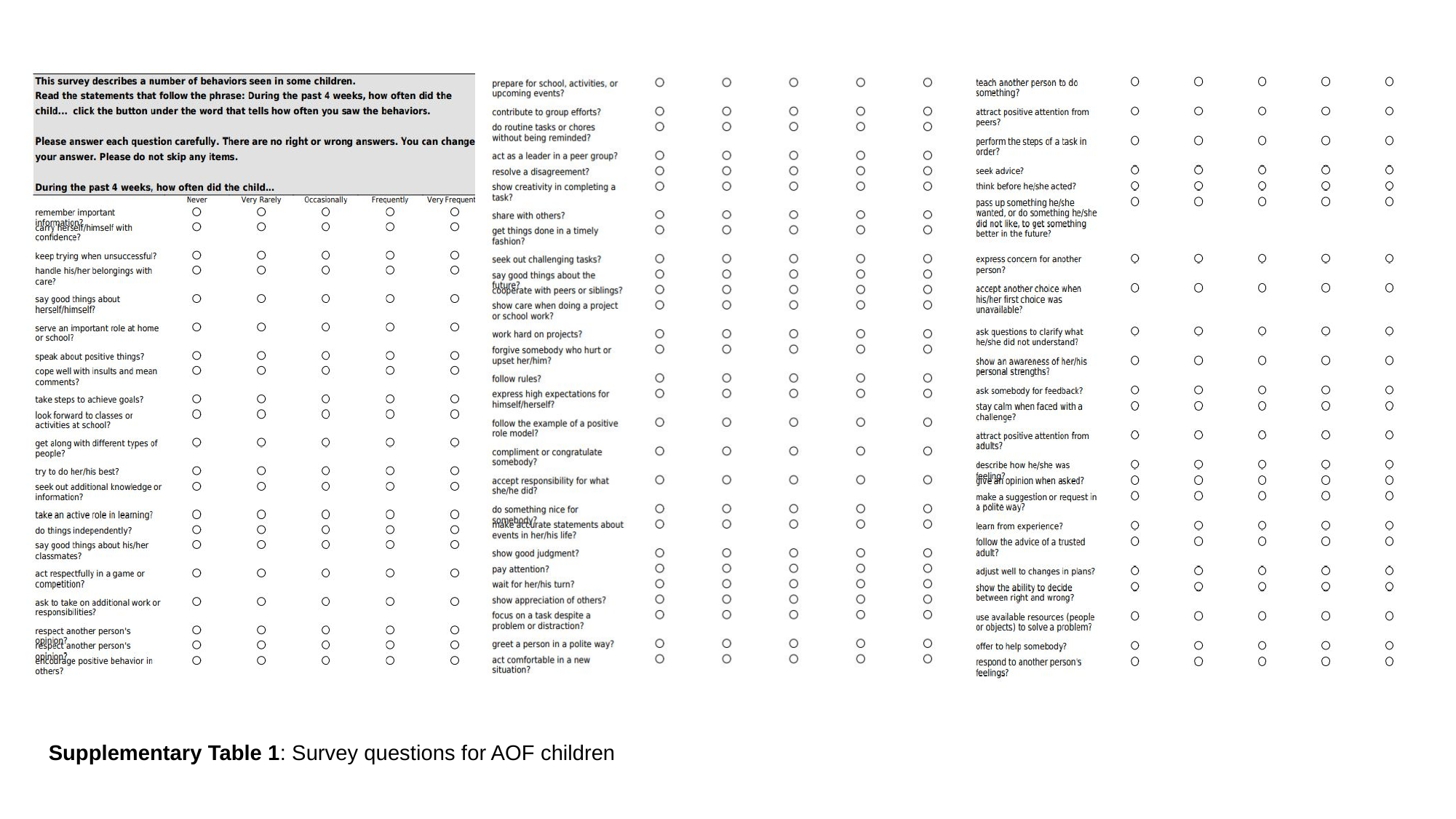

Supplementary Table 1: Survey questions for AOF children

## Slide 2
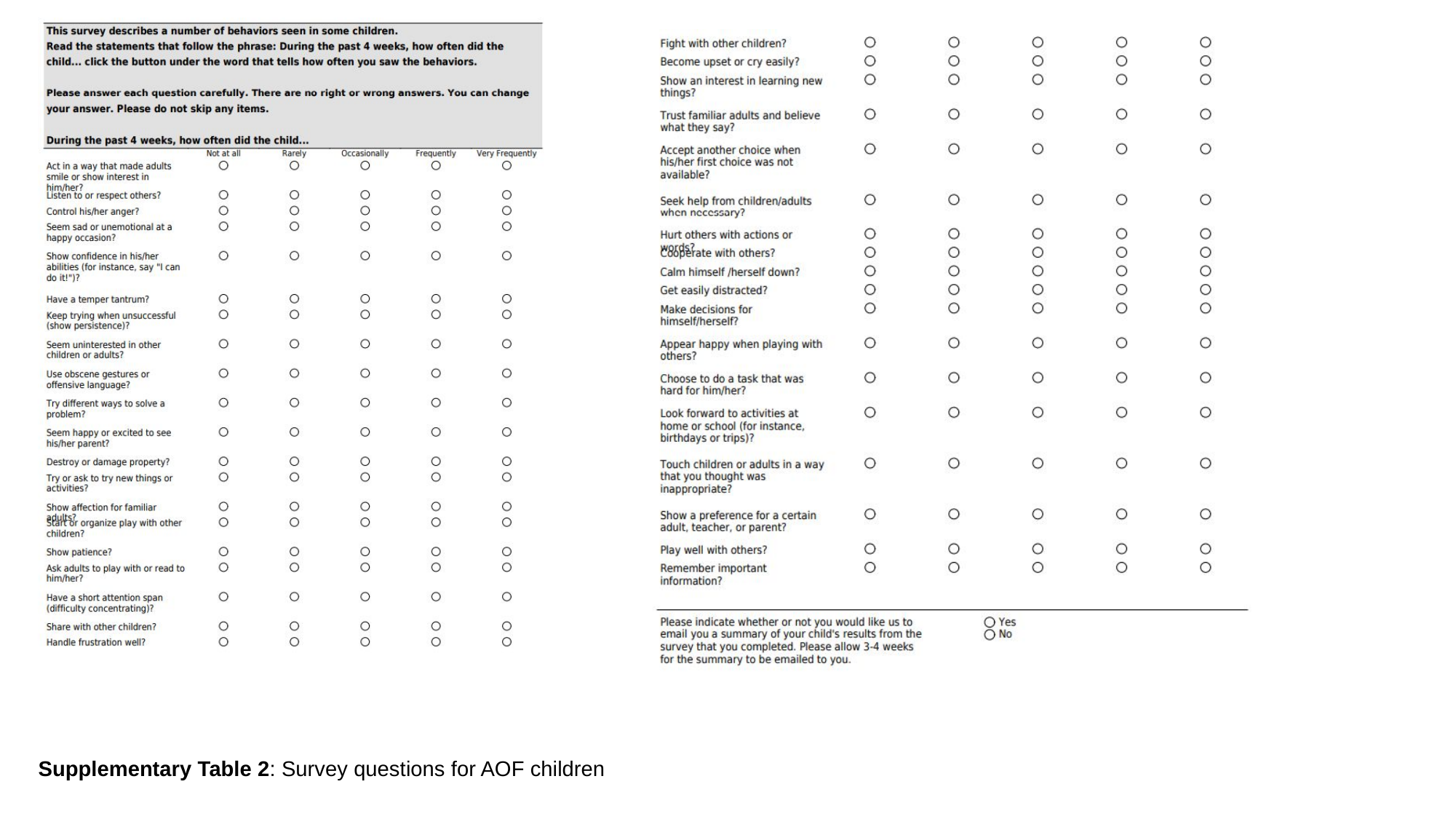

Supplementary Table 2: Survey questions for AOF children

## Slide 3
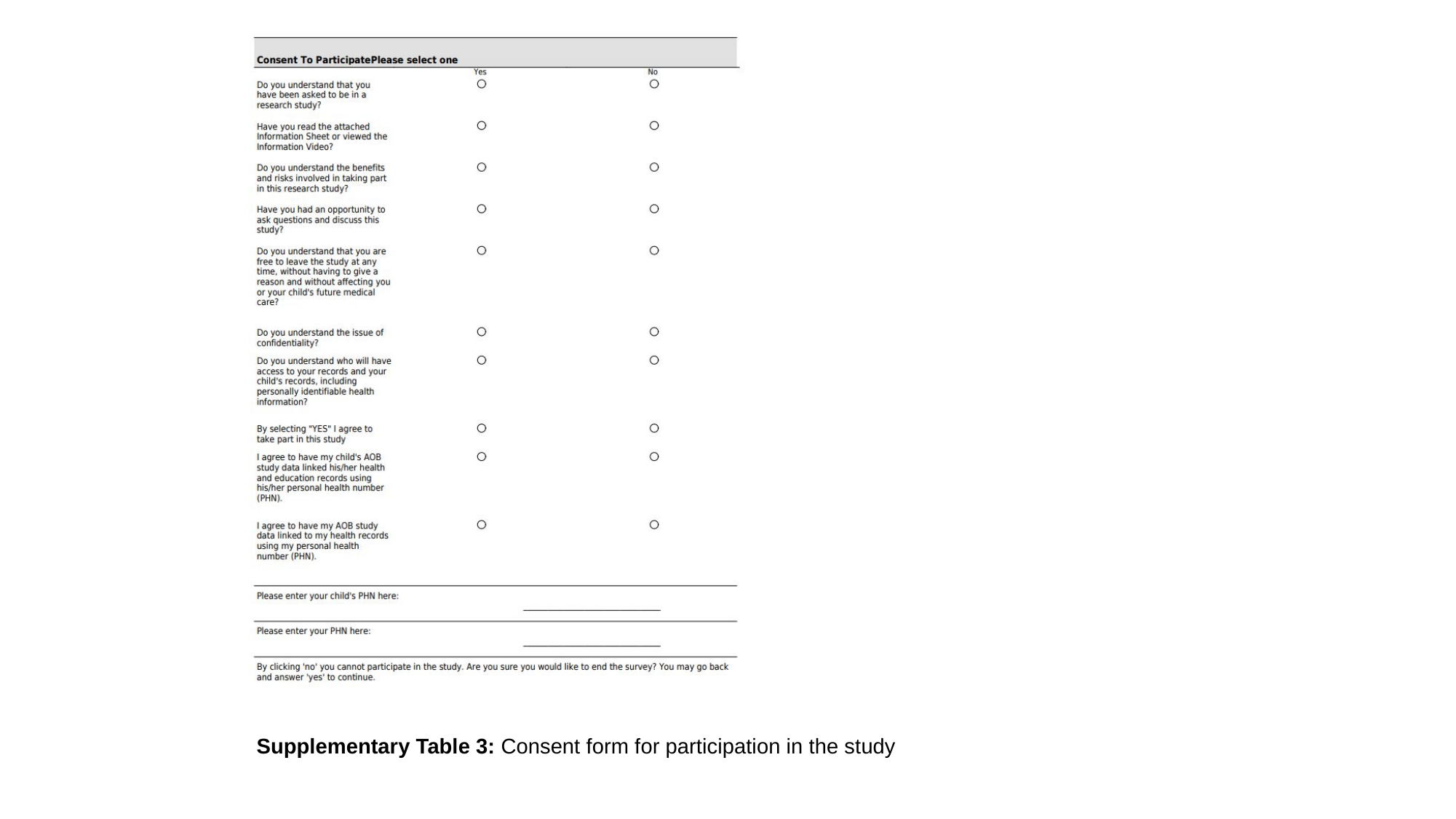

Supplementary Table 3: Consent form for participation in the study

## Slide 4
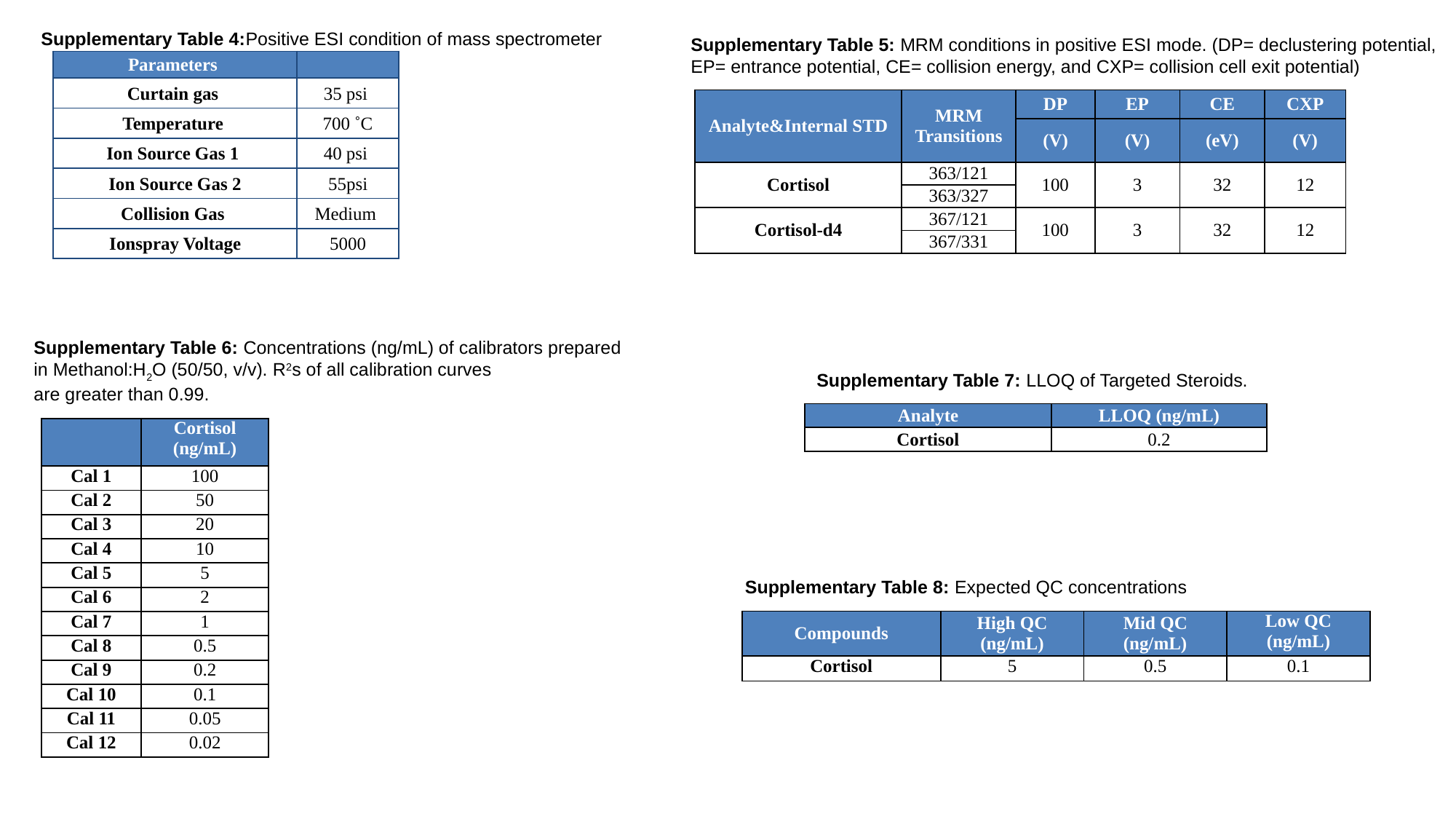

Supplementary Table 4:Positive ESI condition of mass spectrometer
Supplementary Table 5: MRM conditions in positive ESI mode. (DP= declustering potential,
EP= entrance potential, CE= collision energy, and CXP= collision cell exit potential)
| Parameters | |
| --- | --- |
| Curtain gas | 35 psi |
| Temperature | 700 ˚C |
| Ion Source Gas 1 | 40 psi |
| Ion Source Gas 2 | 55psi |
| Collision Gas | Medium |
| Ionspray Voltage | 5000 |
| Analyte&Internal STD | MRM Transitions | DP | EP | CE | CXP |
| --- | --- | --- | --- | --- | --- |
| | | (V) | (V) | (eV) | (V) |
| Cortisol | 363/121 | 100 | 3 | 32 | 12 |
| | 363/327 | | | | |
| Cortisol-d4 | 367/121 | 100 | 3 | 32 | 12 |
| | 367/331 | | | | |
Supplementary Table 6: Concentrations (ng/mL) of calibrators prepared
in Methanol:H2O (50/50, v/v). R2s of all calibration curves
are greater than 0.99.
Supplementary Table 7: LLOQ of Targeted Steroids.
| Analyte | LLOQ (ng/mL) |
| --- | --- |
| Cortisol | 0.2 |
| | Cortisol (ng/mL) |
| --- | --- |
| Cal 1 | 100 |
| Cal 2 | 50 |
| Cal 3 | 20 |
| Cal 4 | 10 |
| Cal 5 | 5 |
| Cal 6 | 2 |
| Cal 7 | 1 |
| Cal 8 | 0.5 |
| Cal 9 | 0.2 |
| Cal 10 | 0.1 |
| Cal 11 | 0.05 |
| Cal 12 | 0.02 |
Supplementary Table 8: Expected QC concentrations
| Compounds | High QC (ng/mL) | Mid QC (ng/mL) | Low QC (ng/mL) |
| --- | --- | --- | --- |
| Cortisol | 5 | 0.5 | 0.1 |
